# New contributions to two ciliate genera (Ciliophora, Heterotrichea) based on morphological and molecular analyses, with description of a new *Gruberia* species

**DOI:** 10.1186/s12866-020-01879-4

**Published:** 2020-10-02

**Authors:** Yong Chi, Yuqing Li, Qianqian Zhang, Mingzhen Ma, Alan Warren, Xiangrui Chen, Weibo Song

**Affiliations:** 1 Institute of Evolution and Marine Biodiversity, and College of Fisheries, Ocean University of China, Qingdao, 266003 China; 2 Yantai Institute of Coastal Zone Research, Chinese Academy of Sciences, Yantai, 264003 China; 3 Center for Ocean Mega-Science, Chinese Academy of Sciences, 7 Nanhai Road, Qingdao, 266071 China; 4 Department of Life Sciences, Natural History Museum, London, SW7 5BD UK; 5 School of Marine Sciences, Ningbo University, Ningbo, 315211 China

**Keywords:** Heterotrichs, Morphology, Phylogeny, SSU rDNA

## Abstract

**Background:**

Heterotrichous ciliates are common members of microeukaryote communities which play important roles in both the transfer of material and the flow of energy in aquatic food webs. This group has been known for over two centuries due to their large body size and cosmopolitan distribution. Nevertheless, species identification and phylogenetic relationships of heterotrichs remain challenging due to the lack of accurate morphological information and insufficient molecular data.

**Results:**

The morphology and phylogeny of two heterotrichous ciliates, namely *Gruberia foissneri* spec. nov. and *Linostomella vorticella* (Ehrenberg, 1833) Aescht in Foissner et al., 1999, were studied using rigorous methods (living morphology, stained preparations, and small subunit rDNA sequence data). *Gruberia foissneri* spec. nov. is morphologically very similar to *G. uninucleata* Kahl, 1932, however, it can be distinguished from the latter by having more ciliary rows (about 32 vs. about 20) and macronuclear shape (sausage-shaped vs. ellipsoid). Based on a combination of previous and present studies, an improved diagnosis of *L. vorticella* is supplied and several taxonomic anomalies are clarified. In addition, phylogenetic analyses based on SSU rDNA sequence data support the generic assignment of these two species.

**Conclusions:**

Modern ciliate taxonomy should be performed by means of detailed living observation, stained preparations and molecular information. For those species that have been reported in previous studies, it is necessary to provide as much useful information as possible using state-of-the-art methods in order to resolve taxonomic anomalies.

## Background

Members of the ciliate class Heterotrichea Stein, 1859 are found in a wide range of aquatic biotopes. The heterotrichs are characterized by their typically large body size, somatic kineties composed of dikinetids with postciliodesmata and a prominent oral apparatus composed of a paroral membrane and an adoral zone of membranelles [1, 2]. According to the two latest works on the classification of heterotrichs [3, 4], the class Heterotrichea contains ten families and about 58 genera, several of which are well-known, e.g., *Condylostoma* Bory de St. Vincent, 1824, *Spirostomum* Ehrenberg, 1834, and *Stentor* Oken, 1815. *Gruberia* Kahl, 1932 is rarely reported and has only three valid species: *G. binucleata* Dragesco, 1960, *G. lanceolata* (Gruber, 1884) Kahl, 1932, and *G. uninucleata* Kahl, 1932 [5, 6]. Of these, only *G. lanceolata* has been investigated using modern methods while its congeners remain insufficiently described [5, 6].

The genus *Linostomella* Aescht in Foissner et al., 1999 is monotypic and classified within the family Condylostomatidae Kahl in Doflein and Reichenow, 1929. The type species, *L. vorticella*, was first reported by Ehrenberg [7] as *Bursaria vorticella* due to the similarity of its body shape with the colpodid *B. truncatella*. Dujardin [8] doubted Ehrenberg’s classification and transferred this species to the heterotrich genus *Condylostoma* because of it is holotrichous somatic ciliation and the conspicuous, spiraled adoral zone of membranelles. More than a century later, Jankowski [9] established the genus *Linostoma* for this species because it has no frontal cirrus/cirri, which is a diagnostic characteristics of *Condylostoma*. Subsequently, Aescht [10] recognized that *Linostoma* is a homonym and re-named it *Linostomella*. Recently, Rossi et al. [11] reported the molecular phylogenetic position of this genus.

**Fig. 1:**
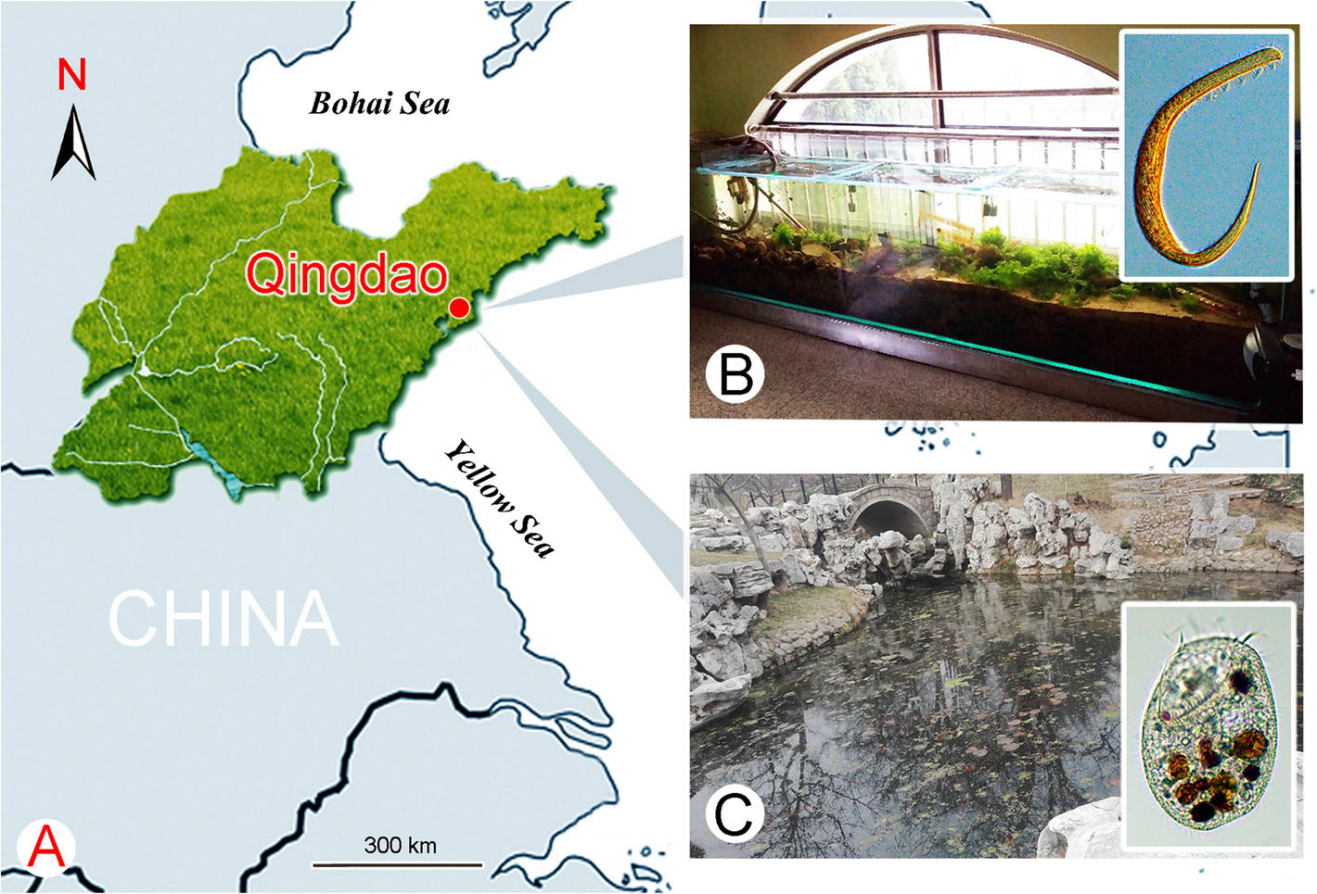
Geographical location of Qingdao and photographs of the sampling sites. **a,** Portion of the map of China, showing location of Qingdao. **b,** The seawater aquarium from which *Gruberia foissneri* spec. nov. was isolated. **c,** The freshwater pond from which *Linostomella vorticella* was isolated

In the present study, two heterotrich species, namely *Gruberia foissneri* spec. nov. and *Linostomella vorticella*, were isolated in Qingdao, China (Fig. 1), giving the opportunity to investigate their taxonomy and phylogeny based on both morphological and molecular data.

## Results

**Zoobank registration.**

urn:lsid:zoobank.org:pub:6D18CFB8-D987-4825-9BA6-72A748AF29B4.

**Family Gruberiidae Shazib et al., 2014.**

**Genus** ***Gruberia*** **Kahl, 1932.**

**Fig. 2:**
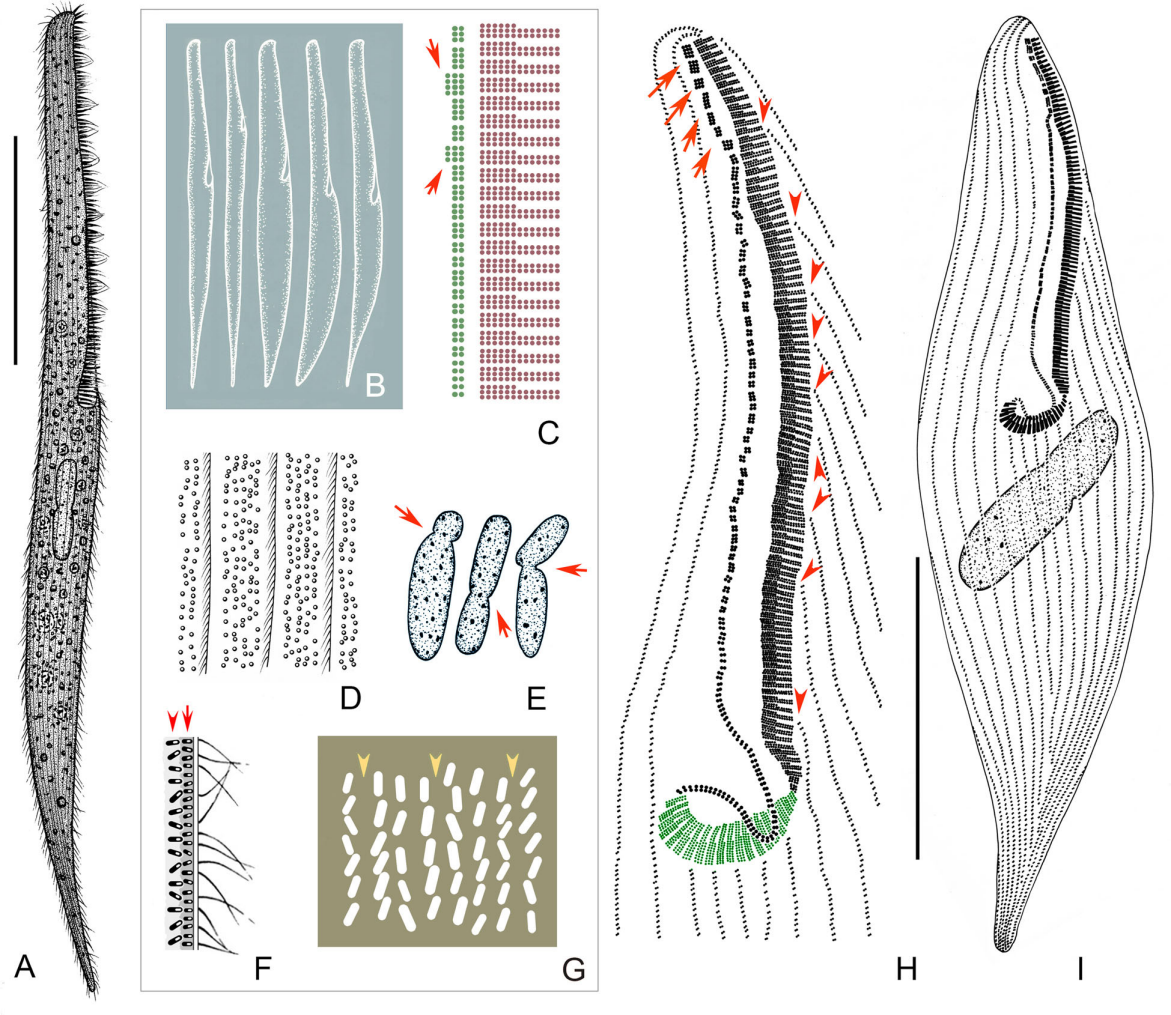
Schematic drawings of *Gruberia foissneri* spec. nov. from life (**a**, **b**, **d**, **f**, **g**) and after protargol staining (**c**, **e**, **h**, **i**). **a,** Right-lateral view of a typical individual. **b,** Various individuals to show different body shapes and ratios of buccal length to body length. **c,** Pattern of the adoral zone of membranelles (red) and paroral membrane (green), arrows show fragments with three rows of kinetosomes. **d,** Cortical granules distributed between the ciliary rows. **e,** Various macronuclear shapes, arrows mark the contracted regions. **f,** Schematic drawing of a tangential section of the cortex, arrow marks the cortical granules, arrowhead indicates the rod-shaped mitochondria (?). **g,** Rod-shaped mitochondria (?) regularly arranged underneath cortex, arrowheads indicate the position of somatic kineties. **h,** Schematic drawing of the adoral membranelles and paroral membrane, green indicates adoral membranelles that enter the oral opening, arrows mark the anterior fragments consisting of two or three rows of kinetosomes, arrowheads show the shortened somatic kineties along the left margin of the adoral zone of membranelles. **i,** Ventral view to show the infraciliature and sausage-shaped macronucleus. Scale bars = 135 μm (**a**), 110 μm (**i**)

**Fig. 3:**
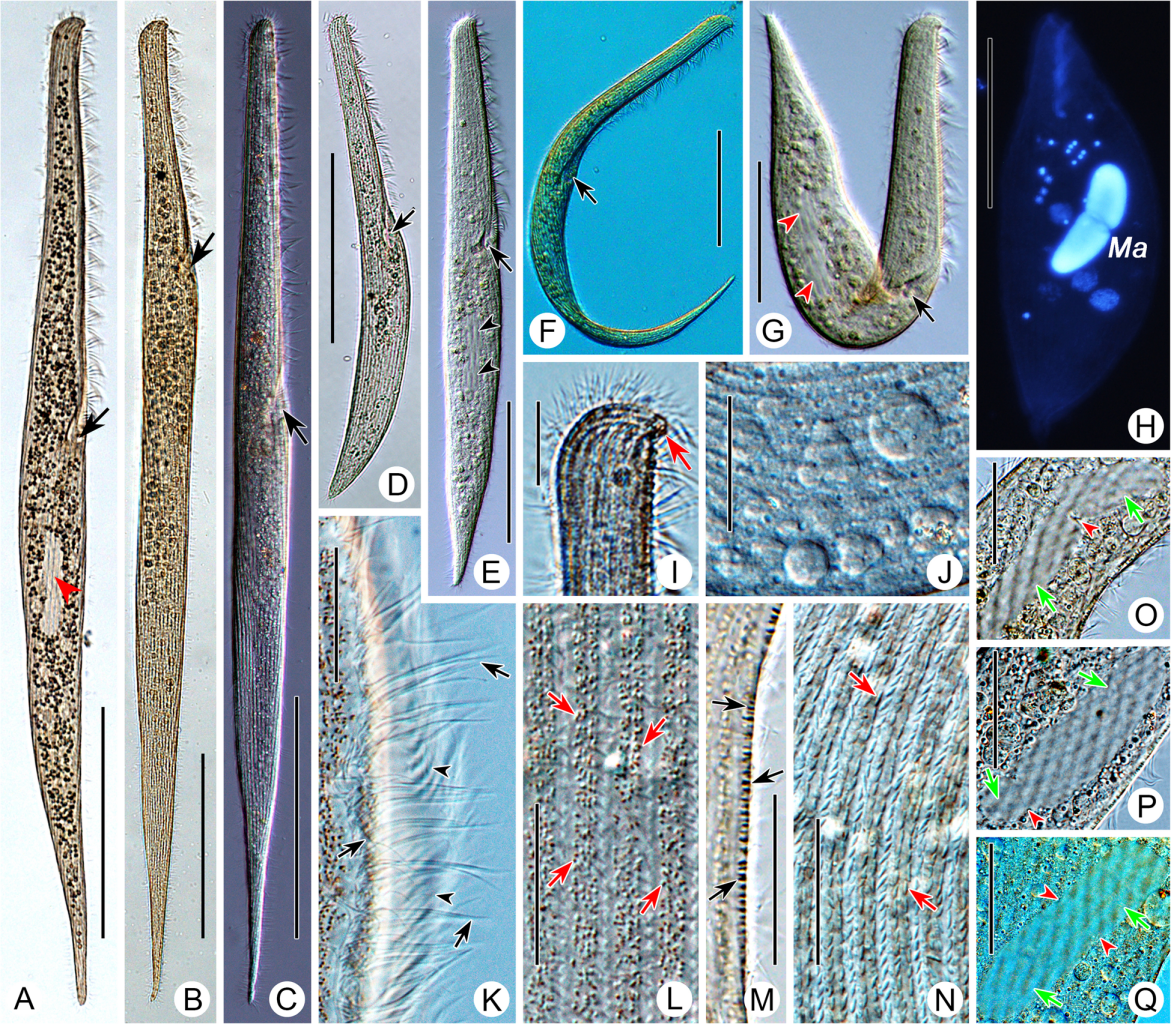
Photomicrographs of *Gruberia foissneri* spec. nov. from life (**a**–**g**, **i**–**q**) and after Hoechst 33342 staining (**h**). **a–g,** General right-lateral views to show the different body shapes and ratios of buccal length to body length, arrows mark the cytopharynx, arrowheads indicate the macronucleus. **h,** Hoechst 33342-stained individual, to show the macronucleus. **i,** Right lateral view of anterior end of cell, arrow marks the rostral apex. **j,** Cytoplasm filled with many empty vacuoles. **k,** Details of adoral zone, arrows mark the conspicuous cilia of the paroral membrane, arrowheads indicate the adoral zone of membranelles. **l,** Cortical granules (arrows) arranged in 3–5 irregular lines between adjacent somatic kineties. **m,** Tangential section of the cell to show the thick cortex, arrows mark the rod-shaped cortical granules. **n,** Rod-shaped mitochondria (?) (arrows) under the cortex. **o–q,** Various macronucleus shapes (arrows), arrowheads indicate the contracted region. Abbreviation: Ma, macronucleus. Scale bars = 150 μm (**a**–**d**), 100 μm (**e**, **f**), 75 μm (**g**, **h**), 20 μm (**i**–**q**)

**Fig. 4:**
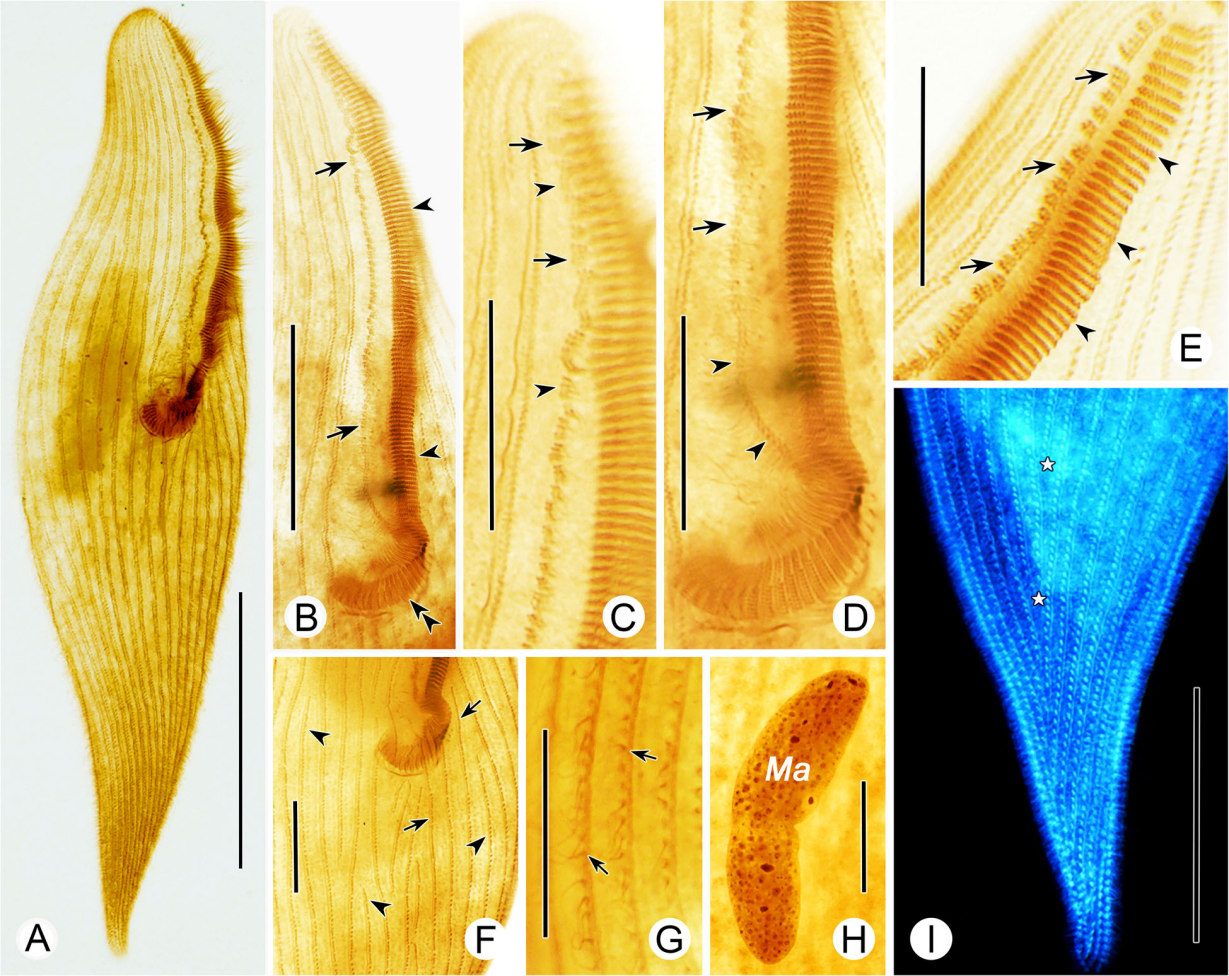
Photomicrographs of *Gruberia foissneri* spec. nov. after protargol staining. **a,** Right-lateral view of a typical specimen. **b,** Detail of oral apparatus, arrows mark the paroral membrane, arrowheads show the adoral zone of membranelles, double arrowheads indicate the cytopharynx. **c,** Enlargement of the anterior part of paroral membrane, arrows indicate the fragments consisting of three lines of kinetosomes, arrowheads mark the fragments consisting of two lines of kinetosomes. **d,** Enlargement of the posterior portion of paroral membrane, arrows mark fragments composed of two rows of kinetosomes, arrowheads indicate the proximal region of the paroral membrane that is not fragmented. **e,** Ventral view of the adoral zone of membranelles (arrowheads) and paroral membrane (arrows). **f,** Ventral view of the ciliary pattern, arrows mark the shortened kineties originating from left margin of adoral zone of membranelles or oral cavity, arrowheads mark the shortened kineties interspersed among bipolar kineties. **g,** Detail of dikinetids, arrows mark ciliated basal body. **h,** Macronucleus. **i,** Ventral view of posterior portion adjusted by the invertible function in Photoshop, asterisks mark a conspicuous suture. Abbreviation: Ma, macronucleus. Scale bars = 100 μm (**a**), 50 μm (**b**, **i**), 30 μm (**c**–**h**)

***Gruberia foissneri*** **spec. nov. (**Figs. 2, 3, 4**,** Table 1**).**

**Table 1:** Morphometric data for *Gruberia foissneri* spec. nov. (*G. foi*) and *Linostomella vorticella* (*L. vor*)

Character	Species	Min	Max	Mean	M	SD	CV	n
Body, length in vivo (μm)	*G. foi*	400	800	560.0	525.0	144.8	25.9	7
	*L. vor*	135	205	175.0	175.0	22.2	12.7	11
Body, width in vivo (μm)	*G. foi*	30	50	39.3	35.0	6.8	17.2	7
	*L. vor*	70	110	93.2	95.0	11.3	12.2	11
Body, length^a^ (μm)	*G. foi*	185	430	325.1	334.0	53.9	16.6	31
	*L. vor*	150	269	205.4	203.0	27.5	13.4	39
Body, width^a^ (μm)	*G. foi*	57	145	87.1	86.0	15.8	18.1	31
	*L. vor*	111	204	154.7	156.0	21.7	14.1	39
Oral area, length in vivo (μm)	*G. foi*	145	295	200.7	195.0	46.2	23.0	7
	*L. vor*	55	110	80.0	85.0	16.5	20.6	11
Oral area, length^a^ (μm)	*G. foi*	72	190	135.7	140.5	27.4	20.2	30
	*L. vor*	68	130	96.9	96.5	16.2	16.7	34
Adoral membranelles, number	*G. foi*	76	174	136.7	141.0	25.8	18.8	26
	*L. vor*	36	51	43.5	44.0	3.7	8.5	34
Somatic kineties, number (including bipolar and shortened somatic kineties)	*G. foi*	25	37	32.4	32.5	3.2	9.9	28
	*L. vor*	37	51	42.4	42.5	3.5	8.2	22
Shortened somatic kineties, number	*G. foi*	9	21	14.4	14.5	3.3	23.2	26
	*L. vor*	11	18	13.5	13.0	2.0	14.7	25
Fragments of paroral membrane, number	*G. foi*	29	75	56.7	55.5	10.9	19.3	24
	*L. vor*	–	–	–	–	–	–	–
Ma nodules, number	*G. foi*	1	1	1.0	1.0	0	0	21
	*L. vor*	5	12	9.0	10.0	1.8	20.4	31
Ma, length (μm)	*G. foi*	68	100	85.0	84.0	10.4	12.2	21
	*L. vor*^b^	12	41	26.8	26.0	7.9	29.5	31
Ma, width (μm)	*G. foi*	19	33	24.8	25.0	3.7	15.0	21
	*L. vor*^b^	9	26	17.1	17.0	3.0	17.7	31

Abbreviations: *CV* Coefficient of variation in %; *M* Median; *Ma* Macronucleus; *Max* Maximum; *Mean* Arithmetic mean; *Min* Minimum; *n* Number of specimens; *SD* standard deviation

^a^ Data based on protargol-stained specimens, ^b^ Macronuclear nodules were selected randomly in each individual, − Data not available

### Diagnosis

Body about 400–800 × 30–50 μm in vivo, slightly contractile, slender with a conspicuously pointed caudal region; macronucleus sausage-shaped; pellicle with rod-shaped, dark-brownish cortical granules and rod-shaped mitochondria (?); 25–37 somatic kineties, several of which are shortened forming a suture near posterior end of body; 76–174 adoral membranelles; paroral membrane fragmented, comprising 29–75 pieces; marine habitat.

### Type locality

A seawater aquarium in the Laboratory of Protozoology (N36°03′45″, E120°19′52″), Qingdao, China. The seawater, stones and sand in the aquarium were collected from Taipingjiao Marine Wetland Park and the Second Beach in Qingdao along with living sea anemones and *Ulva lactuca*. The water temperature was 24 °C and salinity was 30 ppt.

### Type deposition

One protargol-stained slide containing the holotype specimen marked with an ink circle and one slide with paratype specimens are deposited in the Laboratory of Protozoology, Ocean University of China, China, with registration numbers CY201812200101 and CY201812200102. The other two paratype slides are deposited in the Natural History Museum, London, UK, with registration numbers NHMUN 2020.4.6.1 and NHMUN 2020.4.6.2.

### Dedication

We dedicate this new species to Prof. Wilhelm Foissner, Salzburg University, Austria, in recognition of his tremendous contributions to the study of ciliates.

### Gene sequence

The SSU rDNA sequence derived from a single cell isolated from the same population as the holotype is deposited in GenBank (accession number MN783327).

### Description

When fully extended, cell about 400–800 × 30–50 μm in vivo*,* on average about 560 × 40 μm (185–430 × 57–145 μm in protargol-stained specimens) with length to width ratio about 10–18:1. Body flexible and slightly contractile, elliptical in cross-section, anterior end beak-like, posterior part gradually narrows to a pointed end (Fig. 2a, Fig. 3a–e, i). Macronucleus sausage-shaped with an obvious depression (Fig. 2e, Fig. 3o–q, Fig. 4h). Micronucleus difficult to recognize either in vivo or in protargol preparations. Contractile vacuole absent. Pellicle thick with rod-shaped, dark-brownish cortical granules (about 1.2 × 0.5 μm in size) embedded in cortex, forming 3–5 irregular lines between adjacent somatic kineties (Fig. 2d, f, Fig. 3l, m). Mitochondria (?) rod-shaped, about 2.0 × 0.7 μm in size, located underneath cortex forming three or four rows between adjacent ciliary rows (Fig. 2f, g, Fig. 3n). Cytoplasm opaque at low magnification due to numerous small granules and food vacuoles (Fig. 3a–g, j). Locomotion by gliding over substratum.

Twenty-five to 37 somatic kineties composed of dikinetids, only one basal body of each dikinetid bears a cilium (Fig. 2h, i, Fig. 4g). Somatic cilia 5–7 μm long. About 9–21 shortened somatic kineties, most of which originate from left margin of adoral zone of membranelles or oral cavity, remaining ones interspersed among bipolar kineties (Fig. 2h, Fig. 4f). Several shortened kineties form a conspicuous suture on ventral side near posterior end of body (Fig. 2i, Fig. 4i).

Length of oral area relative to body length highly variable, ranging from 25 to 45% (Fig. 2b, Fig. 3a–e). Adoral zone extends from apical end to main body, oral groove slightly curved to right side, twisted in proximal region making a half-turn as it enters the buccal cavity (Fig. 2h, i, Fig. 3a–g, Fig. 4b, d). About 76–174 adoral membranelles, each composed of one short and two long rows of basal bodies (Fig. 2c, h, Fig. 4b, d). Cilia of membranelles 11–16 μm long in vivo. Paroral membrane fragmented into about 29–75 pieces and arranged along right side of adoral zone of membranelles, almost all fragments composed of two rows of kinetosomes except several anterior ones which comprise three rows; paroral membrane conspicuous, comprising two portions: fragmented main portion with each fragment composed of 2–5 pairs of kinetosomes; twisted, unfragmented posterior portion (Fig. 2c, h, i, Fig. 4b–e). Cilia of paroral membrane conspicuous, well-developed, 19–22 μm long in vivo (Fig. 3k).

**Family Condylostomatidae Kahl in Doflein & Reichenow, 1929.**

**Genus** ***Linostomella*** **Aescht in Foissner et al., 1999.**

**Fig. 5:**
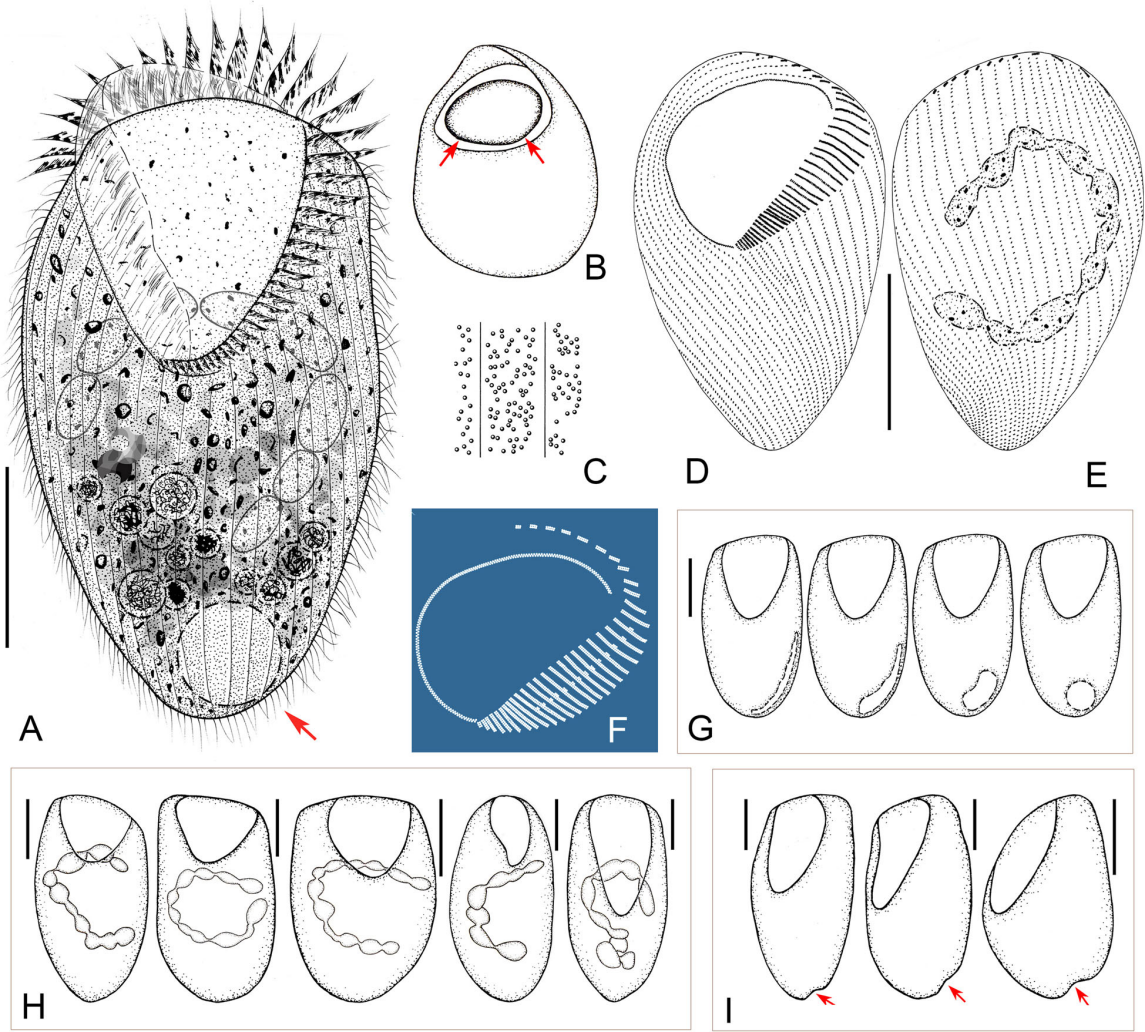
Schematic drawings of *Linostomella vorticella* from life (**a**–**c**, **g**–**i**) and after protargol staining (**d**–**f**). **a,** Ventral view of a typical individual, arrow marks the fully expanded contractile vacuole. **b,** Ventral view of a squashed cell, arrows indicate the oval glabrous protuberance in the buccal cavity. **c,** Cortical granules distributed between the ciliary rows. **d, e,** Ventral (**d**) and dorsal (**e**) views to show the ciliary pattern, oral ciliature and macronucleus. **f,** Schematic drawing of the adoral membranelles and paroral membrane. **g,** To show the diastolic process of the contractile vacuole. **h,** Various individuals to show the different body shapes, ratios of buccal length to body length and distribution of macronuclear nodules. **i,** Left-lateral views of different individuals, arrows mark the depression at posterior end of body. Scale bars = 50 μm (**a**, **g**–**i**), 95 μm (**d**, **e**)

**Fig. 6:**
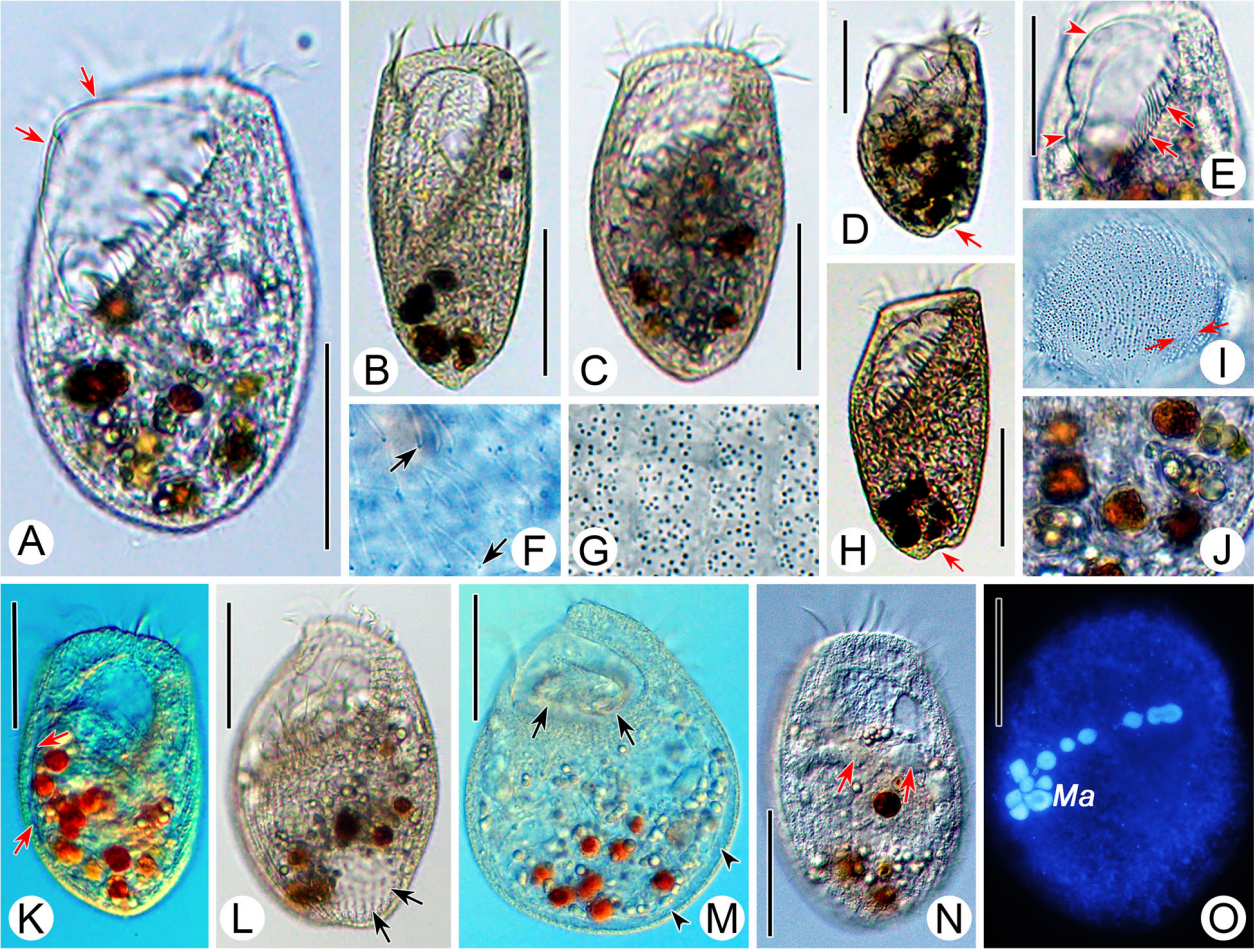
Photomicrographs of *Linostomella vorticella* from life (**a**–**n**) and after Hoechst 33342 staining (**o**). **a–c,** Various individuals to show the different body shapes and ratios of buccal length to body length, arrows mark the prominent paroral membrane. **d, h,** Left-lateral views of different cells, arrows mark the depression at posterior end of body. **e,** Detail of oral area, arrows mark the adoral zone of membranelles, arrowheads show the paroral membrane. **f,** Detail of cilia, arrows denote each basal body bears a cilium. **g,** Tiny cortical granules densely distributed between ciliary rows. **i,** Detail of the glabrous protuberance in oral cavity, arrowheads mark the fiber-like stripes. **j,** Food vacuoles with algae. **k,** Dorsal view of an individual full of food vacuoles, arrows mark the collecting canal. **l,** Contractile vacuole (arrows) near posterior end of body. **m,** Ventral view of a squashed cell, arrows indicate the glabrous protuberance, arrowheads mark different stages in the diastolic process of the contractile vacuole. **N,** Dorsal view of a cell, arrows mark the moniliform macronucleus. **o,** Hoechst 33342-stained individual to show the moniliform macronucleus. Abbreviation: Ma, macronucleus. Scale bars = 60 μm (**a**, **d**, **e**, **k**, **m**, **n**), 90 μm (**b**, **h**), 75 μm (**c**, **l**, **o**)

**Fig. 7:**
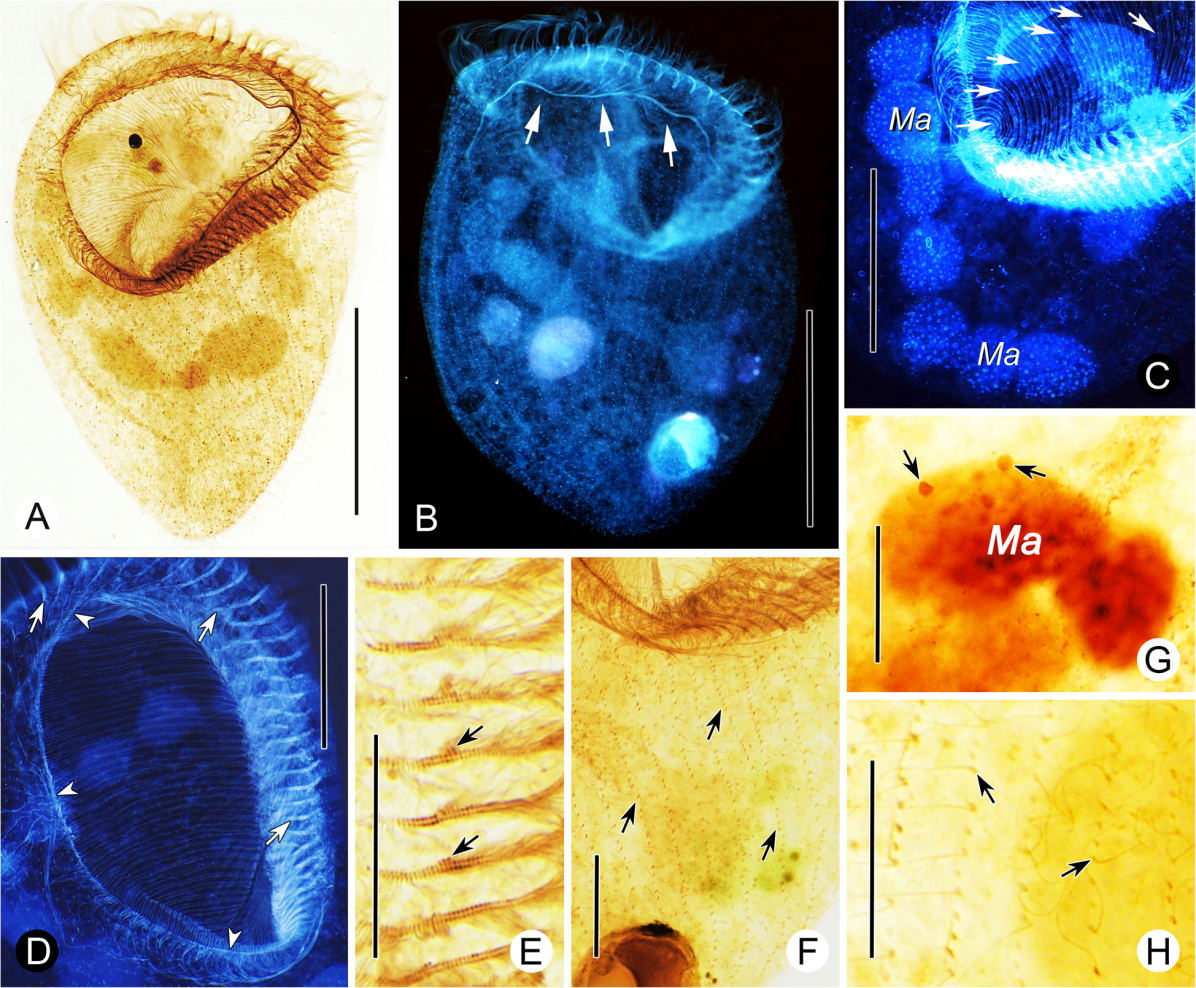
Photomicrographs of *Linostomella vorticella* after protargol staining. **a,** Ventral view of a typical individual. **b–d**, Photomicrographs modified with invertible function in Photoshop. **b,** Dorsal view of a cell, arrows mark the paroral membrane. **c,** Detail of the moniliform macronucleus and conspicuous oral ribs (arrows). **d,** Detail of oral apparatus, arrows indicate the adoral zone of membranelles, arrowheads show the paroral membrane. **e,** Detail of the adoral zone of membranelles, arrows denote some membranelles composed of three rows of basal bodies, one of which is very short. **f,** Detail of somatic kineties, arrows mark the shortened somatic kineties. **g,** Detail of the macronucleus and micronuclei (arrows). **h,** Detail of dikinetids, arrows indicate that only one basal body of each dikinetid bears a cilium. Abbreviation: Ma, macronucleus. Scale bars = 95 μm (**a**, **b**), 50 μm (**c**, **d**), 20 μm (**e**–**h**)

***Linostomella vorticella*** **(Ehrenberg, 1833) Aescht in Foissner et al., 1999 (**Figs. 5, 6, 7**,** Table 1**).**

**Synonyms.**

1833 *Bursaria vorticella* n. sp. – Ehrenberg, Abh dt Akad Wiss 237 (original description without illustration) (present work: Table 3) [7].

1838 *Bursaria vorticella* Ehrenberg, 1833 – Ehrenberg, Infusionsthierchen 326, 327 [Fig. VI] (brief redescription) [24].

1841 *Bursaria vorticella* Ehrenberg – Dujardin, Zoophytes 511 (without morphological description, only simple review of Ehrenberg’s works) [8].

1870 *Condylostoma stagnale* – Wrześniowski, Z wiss Zool 20: 487–489 [Fig. 20] (redescription of living morphology) (present work: Table 3) [12].

1922 *Condylostoma vorticella* (Ehrenberg) Dujardin – Penard, Études Infusoires 201, 202 [Fig. 200] (morphological redescription based on living cell) (present work: Table 3) [13].

1924 *Condylostoma vorticella* (Ehrenberg, 1833) – Fauré-Fremiet, Bull biol Fr Belg 6: 136–139 [Fig. 45] (redescription from life) (present work: Table 3) [14].

1932 *Condylostoma* (*Bursaria*) *vorticella* (Ehrenberg, 1833) – Kahl, Tierwelt Dtl 25: 457 [Figs. 12–14 on page 454, Fig. 28 on page 458] (short revision with simple redescription) (present work: Table 3) [15].

1933 *Condylostoma vorticella* (Ehrenberg) Dujardin 1841 – Wang & Nie, Contr biol Lab Sci Soc China 10: 45–48 [Fig. 36] (redescription of morphology based on living cells) (present work: Table 3) [16].

1967 *Condylostoma vorticella* – Tuffrau, Protistologica 3: 381, 382 [Fig. 7] (brief redescription) [25].

1974 *Condylostoma vorticella* (Ehrenberg) – Pätsch, Arb Inst landw Zool Bienenkd 1: 48, 49 [Fig. 38] (brief redescription, including the infraciliature information) (present work: Table 3) [19].

1978 *Linostoma vorticella* Ehrenberg – Jankowski, Tezisy Dokl zool Inst Akad Nauk SSSR, Jahr 39 (proposal for the establishment of genus *Linostoma*) [9].

1986 *Condylostoma vorticella* Ehrenberg, 1833 – Dragesco & Dragesco-Kernéis, Faune Tropicale 391–393 [Figs. A–D] (simple redescription including infraciliature information) (present work: Table 3) [20].

1991 *Condylostoma vorticella* (Ehrenberg, 1838) – Packroff & Wilbert, Arch Protistenkd 140: 132–134 [Fig. 7] (detailed morphological redescription from life and protargol-stained individuals) (present work: Table 3) [21].

1992 *Linostoma vorticella* (Ehrenberg, 1833) Jankowski, 1978 – Foissner et al., Informationsberichte des Bayer Landesamtes für Wasserwirtschaft 5/92: 390–393 [Figs. 1–14] (diagnosis based on previous reports) (present work: Table 3) [26].

1999 *Linostomella vorticella* (Ehrenberg, 1833) Aescht nov. nom. nov. comb. – Foissner et al., Informationsberichte des Bayerischen Landesamtes für Wasserwirtschaft 3/99: 655–661 [Figs. 1–32] (improved diagnosis provided based on detailed morphological redescription) (present work: Table 3) [22].

2007 *Linostomella vorticella* (Ehrenberg, 1838) – Alekperov et al., Protistology 5: 117, 118 [Fig. 9, Plate 2D on page 114] (simple redescription) (Present work: Table 3) [23].

Prior to the current investigation, *Linostomella vorticella* has been found and reported numerous times, but some details of its morphology remain unknown. Based on both previous and present studies, an improved diagnosis is supplied.

### Improved diagnosis

Cell size in vivo about 90–210 × 70–160 μm; body ovoidal or ellipsoidal with anterior end obliquely truncated; macronucleus moniliform with 2–15 nodules; single contractile vacuole posteriorly positioned with a long collecting canal; cortical granules colorless to dark-gray; about 26–51 somatic kineties; buccal cavity conspicuous with numerous oral ribs; 36–51 adoral membranelles; freshwater and marine habitats.

### Voucher slides

Three voucher slides with protargol-stained specimens are deposited in the Laboratory of Protozoology, Ocean University of China (OUC) with registration numbers: CY2019010501–01, 02, 03.

### Morphological description of the Qingdao population

Cell size 135–205 × 70–110 μm in vivo*,* about 175 × 95 μm on average. Body ovoid in outline with length to width ratio about 1.5–2.0:1 (Fig. 5a, h, Fig. 6a–c). In general, anterior half wider than posterior half, apical end obliquely truncated, posterior end with a slight depression (Fig. 5h, i, Fig. 6d, h). Macronucleus moniliform with 5–12 nodules, located in middle portion of body (Fig. 5a, e, h, Fig. 6n, o, Fig. 7a, c). Micronuclei inconspicuous, closely associated with macronuclear nodules (Fig. 7g). Contractile vacuole in posterior region, varies in shape during diastolic process, with a collecting canal that extends to anterior region of body (Fig. 5a, g, Fig. 6k–m). Pellicle soft and thin with numerous spherical, dark-gray cortical granules (about 0.9 μm in diameter) densely distributed between ciliary rows (Fig. 5c, Fig. 6g). Cytoplasm colorless, invariably filled with numerous globular particles and food vacuoles filled with algae (Fig. 5a, Fig. 6j, k–n). Locomotion by swimming while rotating about main body axis.

Thirty-seven to 51 somatic kineties composed of dikinetids, only one basal body of each dikinetid bears a cilium (Fig. 5d, e, Fig. 6f, Fig. 7h). Somatic cilia 9–12 μm long. About 11–18 ventral kineties are shortened since they originate below buccal cavity; all dorsal kineties extend along complete length of cell (Fig. 5d, e, Fig. 7a, b, f).

Buccal cavity prominent, length about 35–60% of body length, with numerous oral ribs (Fig. 5h, Fig. 6a–c, Fig. 7c, d). Oval glabrous protuberance with fiber-like stripes visible in slightly squashed specimens (Fig. 5b, Fig. 6i, m). Adoral zone of membranelles prominent, composed of 36–51 membranelles, most of which consist of two rows of basal bodies of equal length; several adoral membranelles in middle portion consist of three rows of basal bodies, third row with only two or three basal bodies (Fig. 5d, f, Fig. 7e). Cilia of adoral membranelles 20–30 μm long in vivo. Paroral membrane conspicuous, curved and lies along right margin of buccal cavity, anterior portion curves toward the left side of buccal cavity, posterior portion located near distal end of adoral zone (Fig. 6a, e, Fig. 7b–d).

### Molecular data and phylogenetic analyses

**Fig. 8:**
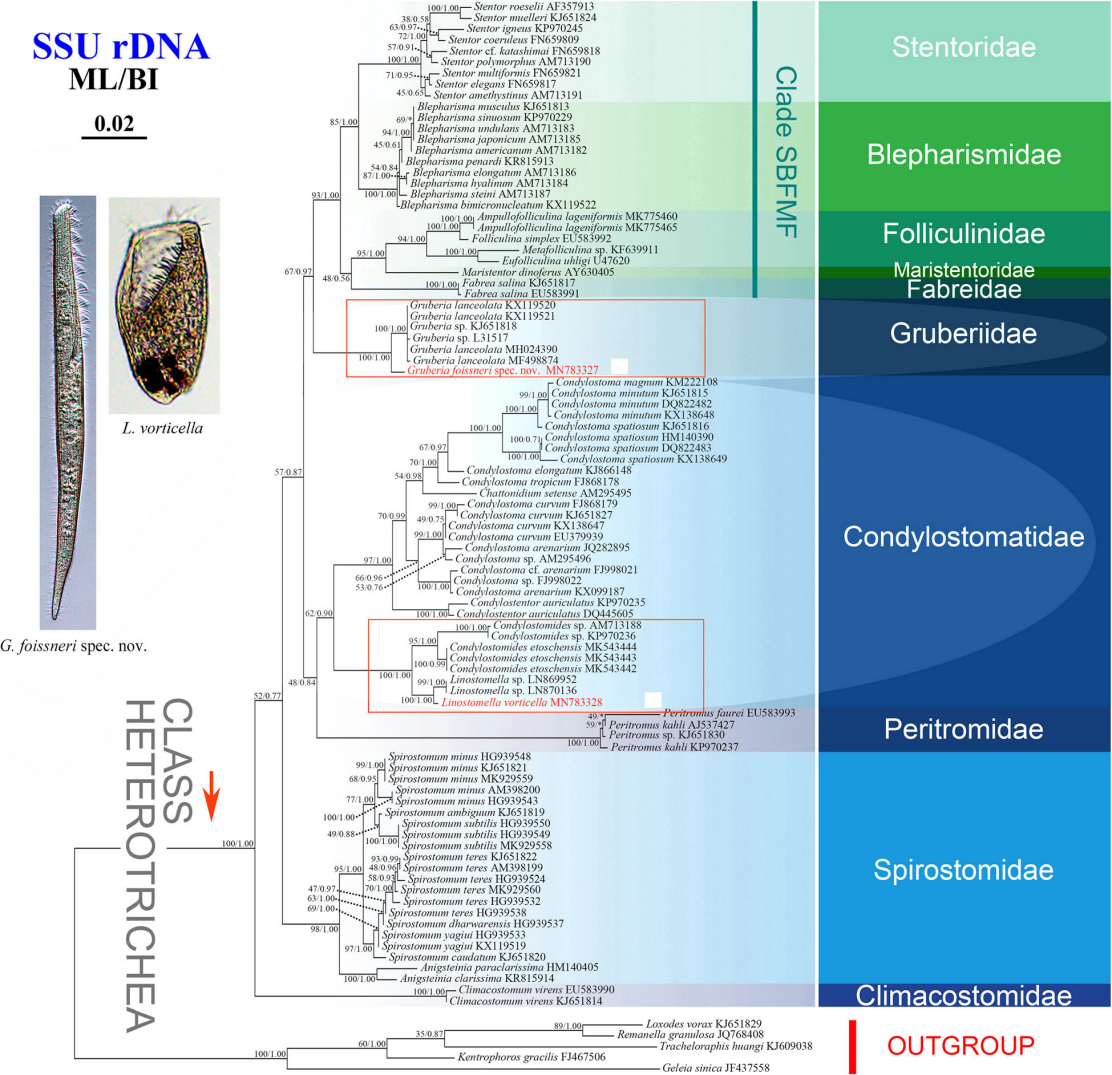
Maximum likelihood (ML) phylogenetic tree inferred from 18S rDNA sequences (91 heterotrichean and 5 karyorelictean taxa). The posterior probabilities from the Bayesian inference (BI) were mapped onto the ML tree. Asterisks indicate a mismatch in branching pattern between the ML and BI trees. The newly sequenced species in this study are shown in red font. The scale bar corresponds to 2 substitutions per 100 nucleotide positions

The two new SSU rDNA sequences obtained in this study were deposited in the GenBank database with lengths, G + C contents, and accession numbers as follows: *Gruberia foissneri* spec. nov., 1627 bp, 46.22%, MN783327; *Linostomella vorticella*, 1683 bp, 46.88%, MN783328. The Maximum likelihood (ML) and Bayesian inference (BI) trees based on SSU rDNA data had nearly identical topologies, therefore only the ML tree is shown with support values from both analyses (Fig. 8).

Seven sequences of *Gruberia* were included in the present analyses, i.e., the newly obtained sequence of *G. foissneri* spec. nov. and six sequences obtained from the GenBank database. These seven sequences form a maximally supported clade (100% ML, 1.00 BI) that represents the family Gruberiidae in the SSU rDNA tree (Fig. 8).

*Linostomella vorticella* and two other *Linostomella* sequences (LN869952, LN870136) cluster together with maximal support (100% ML, 1.00 BI), forming a sister-group to the *Condylostomides* assemblage (100% ML, 1.00 BI). The *Linostomella*-*Condylostomides* clade comprises one of the two sub-clades of the family Condylostomatidae; the other sub-clade contains the genera *Condylostoma*, *Chattonidium*, and *Condylostentor*.

## Discussion

### Comments on *Gruberia foissneri* spec. nov.

The genus *Gruberia* was established by Kahl [15] with *G. uninucleata* as the type species. The morphology of *Gruberia* is similar to that of *Spirostomum* in having an elongated, slightly contractile body and a well-developed peristomial region, although the body of *Gruberia* lacks spiraling or torsion [6, 27]. Seven nominal species of *Gruberia* have been reported: *G. aculeata* Ozaki & Yagiu, 1941, *G. beninensis* Dragesco & Dragesco-Kernéis, 1986, *G. binucleata* Dragesco, 1960, *G. calkinsi* Beltran, 1933, *G. lanceolata* (Gruber, 1884) Kahl, 1932, *G. nematodomorpha* Lepsi, 1965, and *G. uninucleata* Kahl, 1932 [15, 20, 28–32]. In their generic review, Campello-Nunes et al. [5] and Chen et al. [6] synonymized *G. aculeata*, *G. beninensis* and *G. calkinsi* with *G. lanceolata*, and considered *G. nematodomorpha* as a nomen nudum. We accept these decisions and recognize only four valid species, namely *G. uninucleata*, *G. binucleata*, *G. lanceolata* and *G. foissneri* spec. nov.

*Gruberia foissneri* spec. nov. can be easily distinguished from two of its three congeners by its sausage-shaped macronucleus (vs. two oval macronuclei in *G. binucleata* and a moniliform macronucleus in *G. lanceolata*) (Table 2) [5, 6, 29, 30]. In contrast, *G. foissneri* spec. nov. is very similar to *G. uninucleata* which was originally discovered by Kahl [15] from an aquarium in Helgoland, Germany. Kahl [15] described the organism based on living observations as follows: “Gr. 300–650μ; Schlank spindelförmig, im hinteren Drittel gleichmäßig zu einem dünnen Schwanzstachel ausgezogen, der mit kurzkonischer Spitze endigt; 8-10 Reihen auf einer Seite; Ma, ellipsoid” (translation: *size 300–650* *μm; slender spindle-shaped, posterior third evenly narrowed to a thin tail ending with short conical tip; 8–10 ciliary rows on one side; macronucleus, ellipsoid*) (Table 2). Dragesco [33] supplied comprehensive data of a Roscoff population based on living morphology and infraciliature (Table 2). According to these two reports, *G. uninucleata* can be characterized by: (1) cell size about 250–650 μm in vivo; (2) slender body shape with a pointed caudal region; (3) single ellipsoidal macronucleus; (4) about 20 somatic kineties; (5) oral area about 25–33% of body length, with 40–82 adoral membranelles about 70 on average; (6) paroral membrane fragmented, comprising about 23–29 pieces (Table 2). *Gruberia foissneri* spec. nov. is very similar to *G. uninucleata* in the living morphology, however the former can be easily distinguished from the latter by the following characters: (1) number of somatic kineties (25–37, about 32 on average vs. about 20 in *G. uninucleata*); (2) number of adoral zone of membranelles (76–174, about 137 on average vs. 40–82, about 70 on average in *G. uninucleata*); (3) number of paroral membrane fragments (29–75, about 57 on average vs. 23–29 in *G. uninucleata*); (4) macronucleus shape (sausage-shaped with an obvious depression vs. ellipsoidal in *G. uninucleata*).

**Table 2:** Comparison of *Gruberia foissneri* spec. nov. with three congeners

Species	Body size	Peristome length^a^	Number of adoral membranelles	Number of SK (including bipolar and shortened SK)	Number of FPM	Ma number and shape	CV*	Collection site	Reference
*G. foissneri* spec. nov.	400–800 × 30–50	25–45%	76–174	25–37	29–75	Single, sausage-shaped	Absent	A seawater aquarium, Qingdao, China	Present work
*G. uninucleata* (original description)	300–650	25–33%	–	8–10 on one side	–	Single, ellipsoid	Present	An aquarium drain collection box, Helgoland, Germany	Kahl [15]
*G. uninucleata*	250–600	ca. 28%^b^	40–82	16–22	23–29	Single^b^	–	Roscoff, France	Dragesco [33]
*G. binucleata* (original description)	–	–	–	20	–	Two, oval	Present	L’ile Verte, France	Dragesco [29]
*G. lanceolata* (original description)	200	–	–	–	–	Moniliform	–	Genova, Italy	Gruber [30]

Abbreviations: CV* Contractile vacuole; *FPM* Fragments of paroral membrane; *Ma* Macronucleus; *SK* Somatic kineties

^a^ Ratio of oral length to body length, ^b^ Data from drawing or pictures, − Data not available

It is worth noting that Dragesco [34] described a smaller *Gruberia uninucleata* (200 μm on average) based on living observations of a Port-Etienne population. Like the population described by Kahl [15], this population has an ellispoidal macronucleus but possesses about 40 (vs. 8–10 on one side in the population described by Kahl) somatic kineties. In view of the unavailability of key morphological characters and difference in the number of somatic kineties, we suspect that this population may either be conspecific with *Gruberia foissneri* spec. nov. or represent another species. Further studies are needed to test this hypothesis.

### Comments on Linostomella vorticella

*Linostomella vorticella*, which is mainly found in freshwater, was originally reported as *Bursaria vorticella* by Ehrenberg [7]. It was subsequently named *Condylostoma vorticella* (Ehrenberg, 1833) Dujardin and then *Linostoma vorticella* (Ehrenberg, 1833) Jankowski [9, 13]. Aescht [10] reported that *Linostoma* Jankowski, 1978 is a homonym, thus she re-named it *Linostomella*. For nomenclatural purposes the genus and species names should be cited as *Linostomella* Aescht in Foissner et al., 1999 and *Linostomella vorticella* (Ehrenberg, 1833) Aescht in Foissner et al., 1999, respectively [22].

*Linostomella vorticella* resembles *Condylostoma* in having an expansive oral region at the anterior end of the body and a conspicuous paroral membrane, therefore it was for a long time classified in the genus *Condylostoma*. However, *L. vorticella* can be distinguished from *Condylostoma* by the presence of a contractile vacuole (absent in *Condylostoma*), lack of frontal cirri (present in *Condylostoma*) and only one kinetosome of each dikinetid bears a cilium (both kinetosomes ciliated in *Condylostoma*) [35–38].

*Linostomella vorticella* was originally reported by Ehrenberg [7] under the name *Bursaria vorticella*. Ehrenberg’s description, however, was rather superficial which made the subsequent re-identification of this organism difficult. According to the original and subsequent investigations, this species should be recognizable by the following characters: (1) body shape spherical to ellipsoidal, posterior end rounded, anterior end always slightly truncated; (2) conspicuous oral cavity that occupies about half the body length; (3) macronucleus moniliform with nodules arranged in a horseshoe-shape or an oblique line; (4) contractile vacuole at the posterior end of the body with a long collecting canal (Table 3). Furthermore, three populations (two from Germany and one from Austria) were investigated using a combination of in vivo observations and histological staining methods and were found to closely resemble the original population [7, 19, 21]. The Qingdao population corresponds closely with the populations from Europe. We therefore believe that its identification as *L. vorticella* is correct.

**Table 3:** Morphometric comparison of *Linostomella vorticella* populations with significant data and doubtful species reported under that name

Body shape	Body length	Body width	Peristome length^a^	Number of adoral membranelles	Number of SK	Number of Ma nodules	Collection site	Reference
ellipsoidal and variable, obliquely truncated at the anterior end, a depression at the posterior end	135–205	70–110	35–60%	36–51	37–51	5–12	A freshwater pond, Qingdao, China	Present work
almost spherical body, large and oblique oral cavity in front	–	–	–	–	–	–	A fire bucket, Berlin, Germany	Ehrenberg [7] (type population)
ovoid body with a broadly rounded rim	210	160	ca. 50%	–	–	8	A polluted pond, Warsaw, Poland	Wrześniowski [12]
ovoid body with broad back and truncated forward	200	–	ca. 50%	–	–	5	Under water lilies, Ariana, Tunisia	Penard [13]
globular, hemispherical or ovoid body with obliquely truncated anterior	100–125	–	ca. 45%^b^	–	–	–	–	Fauré-Fremiet [14]
bag-shaped, truncated in front	100–200	–	ca. 51%^b^	–	–	6–10	Clear pools and ponds	Kahl [15]
ovoid body, large and evenly rounded toward the posterior extremity, truncated at the anterior end	180	120	ca. 50%	–	–	5	Various ponds, Nanjing, China	Wang & Nie [16]
–	–	–	ca. 47%^b^	–	60–70	11	Some ponds, Hungary	Gelei [17]^†^
–	160	–	ca. 56%^b^	19–22	31–34	2–7	A freshwater pond, Mokolo, Cameroon	Dragesco [18]^†^
–	140–170	80–110	ca. 58%^b^	ca. 40	30–38	8–12	Kleikuhle, Husum, Germany	Pätsch [19]
oval body, rounded posteriorly	140–170	–	–	ca .44	30–38	2–12	–	Dragesco & Dragesco-Kernéis [20]
bag-shaped, truncated in front, rounded at the back	170–200	100	ca. 53%^b^	40–50	39–45	5–9	Meerfelder Maares, Rheinland-Pfalz, Germany	Packroff & Wilbert [21]
saccular to ellipsoidal, both ends broadly rounded, ventral anterior half obliquely truncated	100–210	70–160	ca. 50%	40–50	26–45	2–15	Eutrophic pond, Salzburg, Austria	Foissner et al. [22]
ellipsoidal, rounded on anterior and posterior ends	90–160	70–120	ca. 50%	ca. 50	ca. 35	8–12	Aransas National Wildlife Refuge, Texas, the United States	Alekperov et al. [23]^†^

Abbreviations: *Ma* Macronucleus; *SK* Somatic kineties

^†^ Doubtful species, ^a^ Ratio of oral length to body length, ^b^ Data from drawing or pictures, − Data not available

Gelei [17] reported an organism that resembles *L. vorticella* in all key characters except the number of somatic kineties (60–70 vs. 26–51 in *L. vorticella*) (Table 3). Although the description provided by Gelei [17] was brief, the somatic kinety number is an important character in ciliate species circumscription, so we posit that this population may represent a different species of *Linostomella*. Dragesco [18] described an isolate collected from a freshwater pond in Mokolo, Cameroon, which has fewer adoral membranelles (19–22) than *L. vorticella* (36–51) (Table 3). We agree with Foissner et al. [22] that this population either represents a different species or was mis-observed. Alekperov et al. [23] reported a marine population of *L. vorticella* from the Mexican Gulf, the key characters of which are consistent with the freshwater populations from Germany, Austria and Qingdao (Table 3). In general, habitat is an important character for ciliate species circumscription, so further evidence is needed to verify the identity of this marine population.

In addition to the populations discussed above, *L. vorticella* has been reported numerous times (Table 3) [12–16, 20]. However, we cannot make effective comparisons due to insufficient morphological descriptions in these reports.

### Phylogenetic analyses based on SSU rDNA sequences

Based on its fragmented paroral membrane, Shazib et al. [4] separated *Gruberia* from the family Spirostomidae and established the new family Gruberiidae. This assignment is supported by the present phylogenetic analyses, in which *Gruberia* is clearly divergent from the family Spirostomidae. All sequences of *Gruberia* form a clade that is the sister-group of the Stentoridae + Blepharismidae + Folliculinidae + Maristentoridae + Fabreidae clade (‘Clade SBFMF’ in Fig. 8). This is consistent with the findings of previous studies [3–6, 39–41], and supports the scenario proposed by Luo et al. [39], which recognized that only species of ‘Clade SBFMF’ possess hypericin-like pigment granules. It is suggested that these pigment granules probably play important roles in the evolution of the class Heterotrichea, including the separation of *Gruberia* from ‘Clade SBFMF’ [3].

The genus *Linostomella* is most closely related to *Condylostomides* in the SSU rDNA tree which is consistent with the phylogenetic analyses in Rossi et al. [11]. The similarities of these two taxa in terms of habitat (freshwater), body shape (ellipsoidal), oral apparatus (conspicuous buccal cavity with adoral zone membrane on the left and paroral membrane on the right), contractile vacuole (present), and macronuclear shape (moniliform) [22, 42] support their close evolutionary relationship. The monophyletic family Condylostomatidae comprises two clearly separated sub-clades, namely *Linostomella* + *Condylostomides* and *Condylostoma* + *Condylostentor* + *Chattonidium*, which is broadly consistent with the findings of Rossi et al. [11]. We suspect that the separation of these sub-clades is probably related to differences in habitat, members of the former clade inhabiting freshwaters whereas members of the latter clade are marine.

The new sequence of *Linostomella vorticella* differs from the two unspecified *Linostomella* sequences (LN869952, LN870136) by 14 and 9 nucleotides respectively. This finding, combined with descriptions of populations that differ significantly in their morphology, suggests that the genus *Linostomella* may be not be monotypic.

## Conclusions

In the present paper we describe two heterotrich ciliates, *Gruberia foissneri* spec. nov. and *Linostomella vorticella*, collected from Qingdao, China, using an integrative approach as suggested by Warren et al. [43]. Although *G. foissneri* spec. nov. closely resembles *G. uninucleata*, we provide evidence that these are separate species. In addition, an improved diagnosis of *L. vorticella* is supplied based on present and previous descriptions. Based on analyses of its morphology and molecular phylogeny, we posit that the genus *Linostomella* is not monotypic*.*

## Methods

### Sample collection, morphological methods, and identification

*Gruberia foissneri* spec. nov. was collected from the sandy surface of a seawater aquarium in the Laboratory of Protozoology (N36°03′45″, E120°19′52″), Qingdao, China, on 20th December 2018; the water temperature was 24 °C and salinity was 30 ppt (Fig. 1c). *Linostomella vorticella* was isolated from a freshwater pond in Baihuayuan Park (N36°03′53″, E120°20′22″), Qingdao, China, on 5th January 2019; the water temperature was 2 °C (Fig. 1d).

Living cells were randomly selected from the original samples and observed at 100–1000× magnification using both bright field and differential interference contrast microscopy (Olympus BX53; Zeiss AXIO Imager. D2). The protargol staining method of Wilbert [44] was used to reveal the infraciliature. The protargol powder was made according to Pan et al. [45]. The invertible function in Photoshop was used to adjust the photomicrographs of the infraciliature to show the structure more clearly. Hoechst 33342 solution was used to reveal the nuclear apparatus [46]. Counts, measurements, and drawings of stained specimens were made from photomicrographs (Nikon Y-IDT). Terminology and systematics are mainly according to Foissner et al. [22], Lynn [2] and Shazib et al. [4].

### DNA extraction, PCR amplification, and sequencing

A single cell of each species was isolated from the original sample and washed five times with filtered habitat water to remove potential contaminants. Extraction of genomic DNA was performed using the DNeasy Blood & Tissue Kit (QIAGEN, Hilden, Germany) following the manufacturer’s instructions. Q5® Hot Start high-fidelity DNA polymerase (NEB, Ipswich, MA) was used to amplify the SSU rDNA using universal eukaryotic primers 82F (5′-GAAACTGCGAATGGCTC-3′) and 18 s-R (5′-TGATCCTTCTGCAGGTTCACCTAC-3′) [47, 48]. Cycling parameters of touchdown PCR were as follows: 1 cycle of initial denaturation at 98 °C for 30 s, followed by 18 cycles of amplification (98 °C, 10 s; 69–51 °C touchdown, 30 s; 72 °C, 1 min), and another 18 cycles (98 °C, 10 s; 51 °C, 30 s; 72 °C, 1 min), with a final extension of 72 °C for 5 min. PCR products were checked using agarose gel and were sequenced in TSINGKE (Qingdao, China). Sequence fragments were assembled into contigs using Seqman (DNAStar).

### Phylogenetic analyses

A total of 96 taxa were used for phylogenetic analyses, including the two newly sequenced species and 94 sequences obtained from the GenBank database (see Fig. 8 for accession numbers). Five karyorelictean species were used as the outgroup. Sequences were aligned using MUSCLE on the web server GUIDANCE (http://guidance.tau.ac.il/ver2/) with default parameters [49]. Ambiguously aligned regions were excluded before phylogenetic analyses using G-blocks version 0.91b [50, 51]. The final alignment with 1431 characters was used to construct phylogenetic trees. Maximum likelihood (ML) analysis was carried out on the CIPRES Science Gateway [52] using RAxML-HPC2 on XSEDE v8.2.12 [53]. Bayesian inference (BI) analysis was performed with MrBayes version 3.2.6 on XSEDE [54, 55] of the CIPRES Science Gateway. GTR+ I+ G was selected as the best fitting evolutionary model by MrModeltest version 2.2 according to the Akaike Information Criterion (AIC) [56]. Markov chain Monte Carlo simulations were then run with two sets of four chains using the default settings. The chain length for the analysis was 10,000,000 generations with trees sampled every 100 generations. The first 10% of trees were discarded as burn-in. MEGA 5.2 [57] was used to visualize tree topology.

## Data Availability

All data generated or analysed during this study are included in the published article.

## Abbreviations

BI: Bayesian inference
CV: Coefficient of variation in %
CV*: Contractile vacuole
FPM: Fragments of paroral membrane
M: Median
Ma: Macronucleus
Max: Maximum
Mean: Arithmetic mean
Min: Minimum
ML: Maximum likelihood
n: Number of specimens
SD: Standard deviation
SK: Somatic kineties
SSU rDNA: Small subunit rDNA
